# Ethyl 2-[(*N*-meth­oxy-*N*-methyl­carbamo­yl)meth­yl]-1-(phenyl­sulfon­yl)-1*H*-indole-3-carboxyl­ate

**DOI:** 10.1107/S1600536808014979

**Published:** 2008-05-21

**Authors:** G. Chakkaravarthi, V. Dhayalan, A. K. Mohanakrishnan, V. Manivannan

**Affiliations:** a Department of Physics, CPCL Polytechnic College, Chennai 600 068, India; b Department of Organic Chemistry, University of Madras, Guindy Campus, Chennai 600 025, India; c Department of Physics, Presidency College, Chennai 600 005, India

## Abstract

In the title compound, C_21_H_22_N_2_O_6_S, the phenyl ring forms a dihedral angle of 83.17 (7)° with the indole ring system. The methyl group of the ester unit is disordered over two positions with site occupancies of 0.635 (6) and 0.365 (6). In the crystal structure, weak intra­molecular C—H⋯O inter­actions and inter­molecular C—H⋯O, C—H⋯N and C—H⋯π inter­actions are observed.

## Related literature

For biological activity, see: Merck (1973 ▶, 1974 ▶); Hendi & Basangoudar (1981 ▶); Kolocouris *et al.* (1994 ▶); Uchida *et al.* (1989 ▶); Shaaban *et al.* (1977 ▶). For the structures of closely related compounds, see: Chakkaravarthi *et al.* (2007 ▶, 2008 ▶).
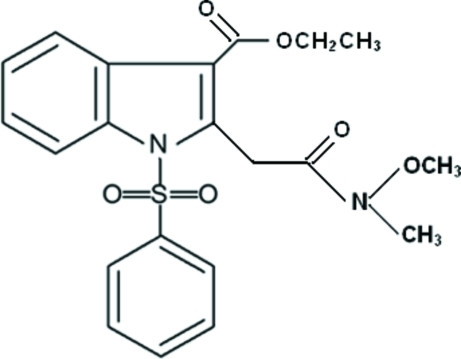


## Experimental

### 

#### Crystal data

C_21_H_22_N_2_O_6_S
*M* *_r_* = 430.47
Monoclinic, 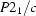

*a* = 8.5827 (3) Å
*b* = 11.0783 (5) Å
*c* = 21.7433 (8) Å
β = 97.091 (2)°
*V* = 2051.58 (14) Å^3^
*Z* = 4
Mo *K*α radiation
μ = 0.20 mm^−1^
*T* = 295 (2) K
0.30 × 0.20 × 0.20 mm

#### Data collection

Bruker Kappa APEX2 diffractometer
Absorption correction: multi-scan (*SADABS*; Sheldrick, 1996 ▶) *T*_min_ = 0.914, *T*_max_ = 0.961
29808 measured reflections
7666 independent reflections
3947 reflections with *I* > 2σ(*I*)
*R*_int_ = 0.029

#### Refinement

*R*[*F* ^2^ > 2σ(*F* ^2^)] = 0.064
*wR*(*F* ^2^) = 0.238
*S* = 1.04
7666 reflections
285 parameters
20 restraints
H-atom parameters constrained
Δρ_max_ = 0.82 e Å^−3^
Δρ_min_ = −0.62 e Å^−3^

### 

Data collection: *APEX2* (Bruker, 2004 ▶); cell refinement: *APEX2*; data reduction: *APEX2*; program(s) used to solve structure: *SHELXS97* (Sheldrick, 2008 ▶); program(s) used to refine structure: *SHELXL97* (Sheldrick, 2008 ▶); molecular graphics: *PLATON* (Spek, 2003 ▶); software used to prepare material for publication: *SHELXL97*.

## Supplementary Material

Crystal structure: contains datablocks I, global. DOI: 10.1107/S1600536808014979/is2294sup1.cif

Structure factors: contains datablocks I. DOI: 10.1107/S1600536808014979/is2294Isup2.hkl

Additional supplementary materials:  crystallographic information; 3D view; checkCIF report

## Figures and Tables

**Table 1:** Hydrogen-bond geometry (Å, °)

*D*—H⋯*A*	*D*—H	H⋯*A*	*D*⋯*A*	*D*—H⋯*A*
C2—H2⋯O2	0.93	2.54	2.896 (3)	103
C6—H6⋯O3	0.93	2.37	3.218 (3)	152
C10—H10⋯O6	0.93	2.46	2.969 (4)	114
C13—H13⋯O2	0.93	2.51	3.039 (4)	117
C15—H15*A*⋯O5	0.97	2.39	2.844 (3)	108
C15—H15*B*⋯O1	0.97	2.17	2.847 (3)	126
C17—H17*B*⋯O5^i^	0.96	2.47	3.420 (3)	173
C20—H20*C*⋯N1^ii^	0.97	2.45	3.351 (4)	155
C20—H20*C*⋯O2^ii^	0.97	2.53	3.343 (4)	142
C21*A*—H21*F*⋯*Cg*^iii^	0.96	2.80	3.196 (13)	105

Symmetry codes: (i) 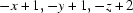; (ii) 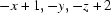; (iii) 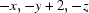. *Cg* is the centroid of the C1–C6 phenyl ring.

**Fig. 1.**
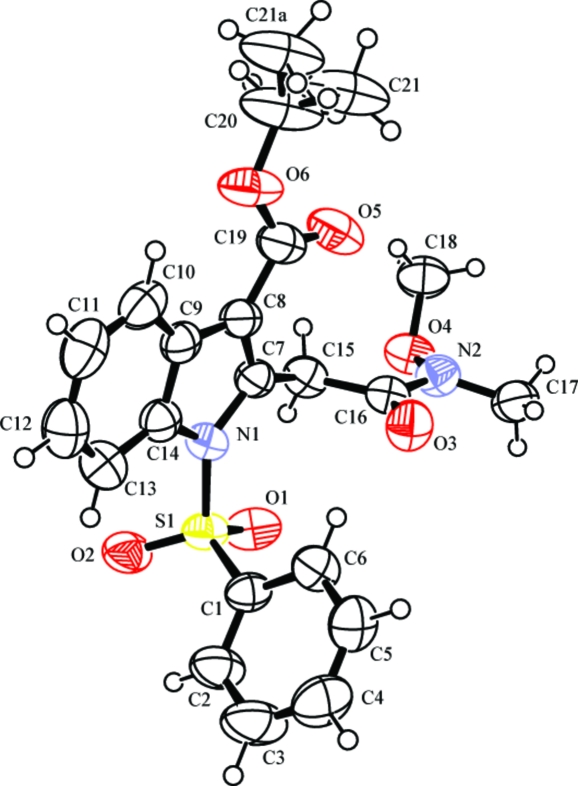
The molecular structure of (I), with atom labels and 50% probability displacement ellipsoids for non-H atoms.

**Fig. 2.**
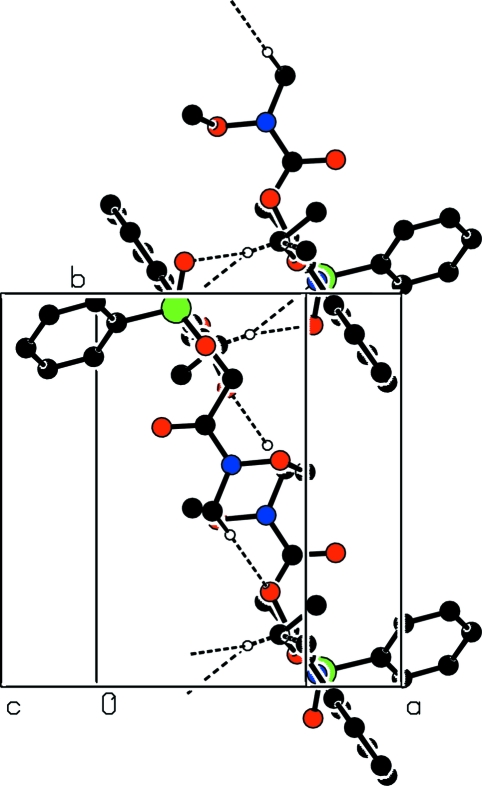
The packing of (I), viewed down the c axis. Intermolecular Hydrogen bonds are shown as dashed lines. H atoms not involved in hydrogen bonding have been omitted.

## supplementary crystallographic information

### Comment

In continuation of our studies of indole derivatives, which are found to possess antihypertensive (Merck, 1973), muscle-relaxant (Hendi & Basangoudar, 1981), antiviral (Kolocouris *et al.*, 1994) antiulcer (Uchida *et al.*, 1989) and analgesic (Shaaban *et al.*, 1977) activities, we determined the crystal structure of the title compound, (I). The geometric parameters of the molecule of (I) (Fig. 1) agree well with those reported for similar structures (Chakkaravarthi *et al.*, 2007, 2008).

The plane of phenyl ring forms 83.17 (7)° with the indole ring system. The plane of N1—S1—C1 forms the dihedral angles of 39.36 (9)° and 69.28 (9)°, respectively, with the phenyl ring and the indole ring. The carboxylate group and *N*-methoxy-*N*-methylcarbamide group are approximately orthogonal to each other [dihedral angle 88.45 (1)°] and makes the dihedral angles of 18.61 (14)° and 84.81 (7)°, respectively, with the indole ring. The sum of bond angles around N1 (359.69°) indicates that N1 is *sp*^2^-hybridized. The torsion angles O1—S1—N1—C7 and O2—S1—N1—C14 [-9.9 (2)° and 48.3 (2)°, respectively] indicate the *syn* conformation of the sulfonyl moiety.

The methyl C atom of the ester group is disordered over two positions with occupancies of 0.635 (6) and 0.365 (6). The molecular structure is stabilized by weak intramolecular C—H···O interactions and the crystal packing (Fig. 2) exhibits weak intermolecular C—H···O, C—H···N interactions and a C—H···π interaction involving the ring C1—C6 (centroid *Cg*) (Table 1).

### Experimental

CO gas was passed through the stirred solution of Ethyl-1-phenylsulfonyl-2-bromomethylindole-3-carboxylate (5.0 g, 11.84 mmol) in CH_3_CN (60 ml). To this, PdCl_2_ (200 mg, 1.12 mmol) and an *in situ* prepared HN(OMe)Me solution [HCl.HN(OMe)Me (230 mg, 23.71 mmol) in CH_3_CN (30 ml)], K_2_CO_3_ (3.27 g, 23.69 mmol) and H_2_O (0.5 ml) were added, stirred for 1 h and filtered] were added. Usual work up followed by evaporation of solvent to give sticky product and the crude product was purified by column chromatography (silica gel) using hexane and ethyl acetate mixture (7:3). Crystals suitable for X-ray analysis were grown by slow evaporation of ethyl acetate solution.

### Refinement

H atoms were positioned geometrically (C—H = 0.93 – 0.97 Å) and refined using riding model, with *U*_iso_(H) = 1.2*U*_eq_(C) or 1.5*U*_eq_(methyl C). The site occupancy factors for disordered C atom was refined as C21 = 0.635 (6) and C21A = 0.365 (6) during anisotropic refinement. The bond distances C20—C21 and C20—C21A were restrained to be 1.5 (3) Å and anisotropic displacement parameters of atoms C20, C21 and C21A were refined with a similar displacement restraint (SIMU) in the final cycles of refinement.

### Crystal data

**Table:**

C_21_H_22_N_2_O_6_S	*F*_000_ = 904
*M**_r_* = 430.47	*D*_x_ = 1.394 Mg m^−^^3^
Monoclinic, *P*2_1_/*c*	Mo *K*α radiation λ = 0.71073 Å
Hall symbol: -P 2ybc	Cell parameters from 8381 reflections
*a* = 8.5827 (3) Å	θ = 2.4–29.8º
*b* = 11.0783 (5) Å	µ = 0.20 mm^−^^1^
*c* = 21.7433 (8) Å	*T* = 295 (2) K
β = 97.091 (2)º	Block, colourless
*V* = 2051.58 (14) Å^3^	0.30 × 0.20 × 0.20 mm
*Z* = 4	

### Data collection

**Table:**

Bruker KappaAPEX2 diffractometer	7666 independent reflections
Radiation source: fine-focus sealed tube	3947 reflections with *I* > 2σ(*I*)
Monochromator: graphite	*R*_int_ = 0.029
*T* = 295(2) K	θ_max_ = 32.9º
ω and φ scans	θ_min_ = 1.9º
Absorption correction: multi-scan(*SADABS*; Sheldrick, 1996)	*h* = −13→12
*T*_min_ = 0.914, *T*_max_ = 0.961	*k* = −16→16
29808 measured reflections	*l* = −33→32

### Refinement

**Table:**

Refinement on *F*^2^	Secondary atom site location: difference Fourier map
Least-squares matrix: full	Hydrogen site location: inferred from neighbouring sites
*R*[*F*^2^ > 2σ(*F*^2^)] = 0.064	H-atom parameters constrained
*wR*(*F*^2^) = 0.238	*w* = 1/[σ^2^(*F*_o_^2^) + (0.1138*P*)^2^ + 0.5762*P*] where *P* = (*F*_o_^2^ + 2*F*_c_^2^)/3
*S* = 1.04	(Δ/σ)_max_ < 0.001
7666 reflections	Δρ_max_ = 0.82 e Å^−^^3^
285 parameters	Δρ_min_ = −0.62 e Å^−^^3^
20 restraints	Extinction correction: none
Primary atom site location: structure-invariant direct methods	

### Fractional atomic coordinates and isotropic or equivalent isotropic displacement parameters (Å^2^)

**Table:**

	*x*	*y*	*z*	*U*_iso_*/*U*_eq_	Occ. (<1)
S1	0.67495 (6)	0.03757 (6)	0.79732 (2)	0.05333 (19)	
O1	0.5718 (2)	0.1330 (2)	0.77694 (8)	0.0696 (5)	
O2	0.6406 (2)	−0.08033 (19)	0.77500 (8)	0.0702 (5)	
O3	0.7525 (2)	0.34032 (17)	0.89517 (10)	0.0685 (5)	
O4	0.36103 (19)	0.42442 (18)	0.88546 (8)	0.0636 (5)	
O5	0.5791 (3)	0.2410 (2)	1.02766 (10)	0.0887 (7)	
N1	0.6850 (2)	0.03196 (17)	0.87461 (8)	0.0500 (4)	
N2	0.5241 (2)	0.4359 (2)	0.89484 (10)	0.0581 (5)	
C1	0.8659 (2)	0.0746 (2)	0.78344 (10)	0.0515 (5)	
C2	0.9197 (3)	0.0215 (3)	0.73297 (13)	0.0733 (8)	
H2	0.8590	−0.0352	0.7093	0.088*	
C3	1.0668 (4)	0.0543 (4)	0.71811 (17)	0.0957 (11)	
H3	1.1061	0.0188	0.6845	0.115*	
C4	1.1538 (3)	0.1389 (3)	0.75290 (16)	0.0841 (9)	
H4	1.2520	0.1606	0.7426	0.101*	
C5	1.0981 (3)	0.1920 (3)	0.80265 (15)	0.0694 (7)	
H5	1.1588	0.2494	0.8258	0.083*	
C6	0.9522 (3)	0.1609 (2)	0.81871 (12)	0.0602 (6)	
H6	0.9133	0.1971	0.8523	0.072*	
C7	0.6276 (2)	0.1142 (2)	0.91567 (10)	0.0484 (5)	
C8	0.6784 (3)	0.0778 (2)	0.97433 (10)	0.0508 (5)	
C9	0.7702 (3)	−0.0301 (2)	0.97205 (10)	0.0500 (5)	
C10	0.8521 (3)	−0.1040 (3)	1.01676 (12)	0.0647 (7)	
H10	0.8514	−0.0882	1.0587	0.078*	
C11	0.9338 (4)	−0.2009 (3)	0.99756 (16)	0.0754 (8)	
H11	0.9901	−0.2504	1.0269	0.090*	
C12	0.9333 (4)	−0.2257 (3)	0.93565 (16)	0.0764 (8)	
H12	0.9885	−0.2926	0.9242	0.092*	
C13	0.8548 (3)	−0.1556 (2)	0.89043 (14)	0.0668 (7)	
H13	0.8556	−0.1728	0.8486	0.080*	
C14	0.7736 (3)	−0.0572 (2)	0.90994 (11)	0.0515 (5)	
C15	0.5211 (3)	0.2171 (2)	0.89660 (11)	0.0553 (5)	
H15A	0.4437	0.2243	0.9253	0.066*	
H15B	0.4659	0.2011	0.8558	0.066*	
C16	0.6103 (3)	0.3346 (2)	0.89525 (10)	0.0524 (5)	
C17	0.5823 (4)	0.5537 (3)	0.88116 (14)	0.0696 (7)	
H17A	0.6936	0.5570	0.8936	0.104*	
H17B	0.5312	0.6139	0.9034	0.104*	
H17C	0.5610	0.5689	0.8375	0.104*	
C18	0.2965 (4)	0.4541 (3)	0.94111 (15)	0.0785 (8)	
H18A	0.3349	0.3983	0.9732	0.118*	
H18B	0.1841	0.4495	0.9338	0.118*	
H18C	0.3273	0.5346	0.9537	0.118*	
C19	0.6408 (3)	0.1433 (3)	1.02879 (12)	0.0629 (6)	
O6	0.6834 (2)	0.0827 (2)	1.08088 (8)	0.0821 (6)	
C20	0.6451 (4)	0.1324 (5)	1.13690 (14)	0.1112 (13)	
H20A	0.6224	0.0677	1.1645	0.133*	0.635 (6)
H20B	0.5515	0.1816	1.1284	0.133*	0.635 (6)
H20C	0.5409	0.1042	1.1426	0.133*	0.365 (6)
H20D	0.6384	0.2193	1.1316	0.133*	0.365 (6)
C21	0.7741 (6)	0.2069 (7)	1.1677 (3)	0.1115 (15)	0.635 (6)
H21A	0.8666	0.1582	1.1767	0.167*	0.635 (6)
H21B	0.7445	0.2389	1.2056	0.167*	0.635 (6)
H21C	0.7952	0.2722	1.1409	0.167*	0.635 (6)
C21A	0.7493 (10)	0.1083 (14)	1.1950 (3)	0.1106 (15)	0.365 (6)
H21D	0.7271	0.0296	1.2102	0.166*	0.365 (6)
H21E	0.7315	0.1680	1.2254	0.166*	0.365 (6)
H21F	0.8568	0.1118	1.1872	0.166*	0.365 (6)

### Atomic displacement parameters (Å^2^)

**Table:**

	*U*^11^	*U*^22^	*U*^33^	*U*^12^	*U*^13^	*U*^23^
S1	0.0435 (3)	0.0740 (4)	0.0426 (3)	−0.0057 (2)	0.0057 (2)	−0.0026 (2)
O1	0.0498 (9)	0.1027 (14)	0.0557 (9)	0.0104 (9)	0.0048 (7)	0.0120 (9)
O2	0.0692 (11)	0.0873 (13)	0.0553 (10)	−0.0237 (10)	0.0127 (8)	−0.0188 (9)
O3	0.0493 (9)	0.0661 (11)	0.0919 (13)	0.0024 (8)	0.0161 (8)	−0.0005 (10)
O4	0.0513 (9)	0.0826 (12)	0.0564 (9)	0.0145 (8)	0.0052 (7)	−0.0038 (8)
O5	0.1006 (16)	0.0973 (17)	0.0711 (13)	0.0049 (13)	0.0215 (11)	−0.0281 (12)
N1	0.0524 (10)	0.0554 (11)	0.0425 (9)	0.0009 (8)	0.0065 (7)	−0.0044 (8)
N2	0.0512 (10)	0.0622 (12)	0.0624 (12)	0.0070 (9)	0.0126 (9)	0.0021 (9)
C1	0.0411 (10)	0.0663 (14)	0.0475 (11)	−0.0022 (9)	0.0073 (8)	0.0010 (10)
C2	0.0582 (14)	0.100 (2)	0.0646 (15)	−0.0152 (14)	0.0181 (12)	−0.0212 (15)
C3	0.0676 (18)	0.140 (3)	0.086 (2)	−0.015 (2)	0.0361 (16)	−0.026 (2)
C4	0.0518 (14)	0.110 (3)	0.093 (2)	−0.0117 (15)	0.0209 (14)	0.0050 (19)
C5	0.0463 (12)	0.0724 (17)	0.0877 (19)	−0.0074 (11)	0.0009 (12)	−0.0014 (14)
C6	0.0494 (12)	0.0692 (15)	0.0617 (14)	−0.0006 (10)	0.0059 (10)	−0.0083 (11)
C7	0.0442 (10)	0.0533 (12)	0.0487 (11)	−0.0063 (9)	0.0099 (8)	−0.0046 (9)
C8	0.0495 (11)	0.0593 (13)	0.0441 (10)	−0.0119 (9)	0.0079 (8)	−0.0049 (9)
C9	0.0477 (10)	0.0534 (12)	0.0486 (11)	−0.0140 (9)	0.0045 (8)	0.0002 (9)
C10	0.0635 (14)	0.0711 (16)	0.0574 (13)	−0.0178 (12)	−0.0003 (11)	0.0120 (12)
C11	0.0720 (17)	0.0644 (17)	0.086 (2)	−0.0079 (13)	−0.0068 (14)	0.0207 (15)
C12	0.0746 (18)	0.0573 (15)	0.096 (2)	0.0052 (13)	0.0070 (15)	0.0031 (15)
C13	0.0689 (15)	0.0629 (15)	0.0688 (16)	0.0048 (12)	0.0093 (12)	−0.0071 (12)
C14	0.0495 (11)	0.0527 (12)	0.0522 (12)	−0.0051 (9)	0.0052 (9)	0.0000 (9)
C15	0.0435 (10)	0.0647 (14)	0.0580 (13)	0.0024 (10)	0.0067 (9)	−0.0059 (11)
C16	0.0504 (11)	0.0612 (13)	0.0462 (11)	0.0039 (10)	0.0080 (8)	−0.0028 (10)
C17	0.0807 (18)	0.0645 (16)	0.0678 (16)	0.0088 (13)	0.0265 (13)	0.0075 (12)
C18	0.0718 (17)	0.091 (2)	0.0787 (19)	0.0057 (15)	0.0315 (14)	−0.0056 (16)
C19	0.0539 (13)	0.0821 (18)	0.0542 (13)	−0.0131 (12)	0.0123 (10)	−0.0155 (12)
O6	0.0712 (12)	0.1331 (19)	0.0432 (9)	−0.0085 (12)	0.0115 (8)	−0.0107 (10)
C20	0.0693 (16)	0.209 (4)	0.0573 (15)	0.010 (2)	0.0169 (12)	−0.032 (2)
C21	0.0722 (19)	0.205 (4)	0.0585 (18)	0.007 (2)	0.0138 (15)	−0.036 (2)
C21A	0.071 (2)	0.206 (4)	0.057 (2)	0.008 (2)	0.0164 (17)	−0.034 (2)

### Geometric parameters (Å, °)

**Table:**

S1—O2	1.412 (2)	C10—H10	0.9300
S1—O1	1.415 (2)	C11—C12	1.373 (5)
S1—N1	1.6732 (19)	C11—H11	0.9300
S1—C1	1.751 (2)	C12—C13	1.364 (4)
O3—C16	1.222 (3)	C12—H12	0.9300
O4—N2	1.395 (3)	C13—C14	1.388 (4)
O4—C18	1.430 (3)	C13—H13	0.9300
O5—C19	1.204 (4)	C15—C16	1.513 (3)
N1—C7	1.407 (3)	C15—H15A	0.9700
N1—C14	1.414 (3)	C15—H15B	0.9700
N2—C16	1.343 (3)	C17—H17A	0.9600
N2—C17	1.442 (4)	C17—H17B	0.9600
C1—C2	1.374 (4)	C17—H17C	0.9600
C1—C6	1.383 (3)	C18—H18A	0.9600
C2—C3	1.390 (4)	C18—H18B	0.9600
C2—H2	0.9300	C18—H18C	0.9600
C3—C4	1.366 (5)	C19—O6	1.328 (4)
C3—H3	0.9300	O6—C20	1.412 (3)
C4—C5	1.368 (5)	C20—C21	1.474 (3)
C4—H4	0.9300	C20—C21A	1.479 (3)
C5—C6	1.385 (4)	C20—H20A	0.9700
C5—H5	0.9300	C20—H20B	0.9700
C6—H6	0.9300	C20—H20C	0.9700
C7—C8	1.358 (3)	C20—H20D	0.9700
C7—C15	1.487 (3)	C21—H21A	0.9600
C8—C9	1.435 (3)	C21—H21B	0.9600
C8—C19	1.458 (3)	C21—H21C	0.9600
C9—C14	1.387 (3)	C21A—H21D	0.9600
C9—C10	1.393 (3)	C21A—H21E	0.9600
C10—C11	1.375 (5)	C21A—H21F	0.9600
			
O2—S1—O1	119.21 (13)	C14—C13—H13	121.7
O2—S1—N1	107.07 (11)	C9—C14—C13	122.6 (2)
O1—S1—N1	107.15 (10)	C9—C14—N1	107.6 (2)
O2—S1—C1	108.50 (12)	C13—C14—N1	129.7 (2)
O1—S1—C1	109.49 (12)	C7—C15—C16	111.70 (18)
N1—S1—C1	104.39 (10)	C7—C15—H15A	109.3
N2—O4—C18	110.0 (2)	C16—C15—H15A	109.3
C7—N1—C14	108.36 (18)	C7—C15—H15B	109.3
C7—N1—S1	129.40 (16)	C16—C15—H15B	109.3
C14—N1—S1	121.93 (15)	H15A—C15—H15B	107.9
C16—N2—O4	117.8 (2)	O3—C16—N2	120.4 (2)
C16—N2—C17	123.6 (2)	O3—C16—C15	123.5 (2)
O4—N2—C17	114.8 (2)	N2—C16—C15	116.1 (2)
C2—C1—C6	121.9 (2)	N2—C17—H17A	109.5
C2—C1—S1	116.83 (19)	N2—C17—H17B	109.5
C6—C1—S1	121.00 (18)	H17A—C17—H17B	109.5
C1—C2—C3	118.6 (3)	N2—C17—H17C	109.5
C1—C2—H2	120.7	H17A—C17—H17C	109.5
C3—C2—H2	120.7	H17B—C17—H17C	109.5
C4—C3—C2	120.0 (3)	O4—C18—H18A	109.5
C4—C3—H3	120.0	O4—C18—H18B	109.5
C2—C3—H3	120.0	H18A—C18—H18B	109.5
C3—C4—C5	120.8 (3)	O4—C18—H18C	109.5
C3—C4—H4	119.6	H18A—C18—H18C	109.5
C5—C4—H4	119.6	H18B—C18—H18C	109.5
C4—C5—C6	120.5 (3)	O5—C19—O6	123.1 (3)
C4—C5—H5	119.8	O5—C19—C8	124.9 (3)
C6—C5—H5	119.8	O6—C19—C8	112.1 (3)
C1—C6—C5	118.1 (2)	C19—O6—C20	118.0 (3)
C1—C6—H6	120.9	O6—C20—C21	111.7 (3)
C5—C6—H6	120.9	O6—C20—C21A	119.1 (5)
C8—C7—N1	107.8 (2)	C21—C20—C21A	51.1 (6)
C8—C7—C15	127.3 (2)	O6—C20—H20A	109.3
N1—C7—C15	124.8 (2)	C21—C20—H20A	109.3
C7—C8—C9	109.2 (2)	O6—C20—H20B	109.3
C7—C8—C19	122.5 (2)	C21—C20—H20B	109.3
C9—C8—C19	128.3 (2)	H20A—C20—H20B	108.0
C14—C9—C10	118.8 (2)	O6—C20—H20C	107.5
C14—C9—C8	106.96 (19)	C21A—C20—H20C	107.5
C10—C9—C8	134.2 (2)	O6—C20—H20D	107.5
C11—C10—C9	118.7 (3)	C21A—C20—H20D	107.5
C11—C10—H10	120.7	H20C—C20—H20D	107.0
C9—C10—H10	120.7	C20—C21—H21A	109.5
C12—C11—C10	120.9 (3)	C20—C21—H21B	109.5
C12—C11—H11	119.6	C20—C21—H21C	109.5
C10—C11—H11	119.6	C20—C21A—H21D	109.5
C13—C12—C11	122.3 (3)	C20—C21A—H21E	109.5
C13—C12—H12	118.9	H21D—C21A—H21E	109.5
C11—C12—H12	118.9	C20—C21A—H21F	109.5
C12—C13—C14	116.7 (3)	H21D—C21A—H21F	109.5
C12—C13—H13	121.7	H21E—C21A—H21F	109.5
			
O2—S1—N1—C7	−138.9 (2)	C19—C8—C9—C10	−1.3 (4)
O1—S1—N1—C7	−9.9 (2)	C14—C9—C10—C11	0.3 (3)
C1—S1—N1—C7	106.2 (2)	C8—C9—C10—C11	−177.9 (2)
O2—S1—N1—C14	48.3 (2)	C9—C10—C11—C12	−0.9 (4)
O1—S1—N1—C14	177.31 (18)	C10—C11—C12—C13	0.9 (5)
C1—S1—N1—C14	−66.6 (2)	C11—C12—C13—C14	−0.3 (4)
C18—O4—N2—C16	111.0 (3)	C10—C9—C14—C13	0.3 (3)
C18—O4—N2—C17	−90.2 (3)	C8—C9—C14—C13	178.9 (2)
O2—S1—C1—C2	29.4 (3)	C10—C9—C14—N1	−178.86 (19)
O1—S1—C1—C2	−102.2 (2)	C8—C9—C14—N1	−0.3 (2)
N1—S1—C1—C2	143.3 (2)	C12—C13—C14—C9	−0.3 (4)
O2—S1—C1—C6	−155.9 (2)	C12—C13—C14—N1	178.7 (3)
O1—S1—C1—C6	72.4 (2)	C7—N1—C14—C9	0.2 (2)
N1—S1—C1—C6	−42.0 (2)	S1—N1—C14—C9	174.29 (15)
C6—C1—C2—C3	1.4 (5)	C7—N1—C14—C13	−178.9 (2)
S1—C1—C2—C3	176.0 (3)	S1—N1—C14—C13	−4.8 (3)
C1—C2—C3—C4	−0.8 (6)	C8—C7—C15—C16	85.8 (3)
C2—C3—C4—C5	0.2 (6)	N1—C7—C15—C16	−98.8 (2)
C3—C4—C5—C6	0.0 (5)	O4—N2—C16—O3	170.5 (2)
C2—C1—C6—C5	−1.2 (4)	C17—N2—C16—O3	13.6 (4)
S1—C1—C6—C5	−175.6 (2)	O4—N2—C16—C15	−10.3 (3)
C4—C5—C6—C1	0.5 (4)	C17—N2—C16—C15	−167.1 (2)
C14—N1—C7—C8	0.0 (2)	C7—C15—C16—O3	16.2 (3)
S1—N1—C7—C8	−173.54 (16)	C7—C15—C16—N2	−163.0 (2)
C14—N1—C7—C15	−176.2 (2)	C7—C8—C19—O5	−9.2 (4)
S1—N1—C7—C15	10.3 (3)	C9—C8—C19—O5	170.6 (3)
N1—C7—C8—C9	−0.2 (2)	C7—C8—C19—O6	171.1 (2)
C15—C7—C8—C9	175.9 (2)	C9—C8—C19—O6	−9.1 (3)
N1—C7—C8—C19	179.7 (2)	O5—C19—O6—C20	4.1 (4)
C15—C7—C8—C19	−4.3 (4)	C8—C19—O6—C20	−176.2 (2)
C7—C8—C9—C14	0.3 (2)	C19—O6—C20—C21	−93.0 (5)
C19—C8—C9—C14	−179.6 (2)	C19—O6—C20—C21A	−149.5 (7)
C7—C8—C9—C10	178.6 (2)		

### Hydrogen-bond geometry (Å, °)

**Table:**

*D*—H···*A*	*D*—H	H···*A*	*D*···*A*	*D*—H···*A*
C2—H2···O2	0.93	2.54	2.896 (3)	103
C6—H6···O3	0.93	2.37	3.218 (3)	152
C10—H10···O6	0.93	2.46	2.969 (4)	114
C13—H13···O2	0.93	2.51	3.039 (4)	117
C15—H15A···O5	0.97	2.39	2.844 (3)	108
C15—H15B···O1	0.97	2.17	2.847 (3)	126
C17—H17B···O5^i^	0.96	2.47	3.420 (3)	173
C20—H20C···N1^ii^	0.97	2.45	3.351 (4)	155
C20—H20C···O2^ii^	0.97	2.53	3.343 (4)	142
C21A—H21F···Cg^iii^	0.96	2.80	3.196 (13)	105

Symmetry codes: (i) −*x*+1, −*y*+1, −*z*+2; (ii) −*x*+1, −*y*, −*z*+2; (iii) −*x*, −*y*+2, −*z*.
